# Energy Measurement Characteristics of Electricity Meters with Different Input Configurations Under IEC 61000-4-19-Based Conducted Disturbances

**DOI:** 10.3390/s26092781

**Published:** 2026-04-29

**Authors:** Grzegorz Sadkowski, Andrzej Bień

**Affiliations:** 1 Electricity and Radiation Department, Electrical Power Engineering Measurements Laboratory, Central Office of Measures, ul. Elektoralna 2, 00-139 Warsaw, Poland; 2 AGH Doctoral School, Faculty of Electrical Engineering, Automatics, Computer Science and Biomedical Engineering, AGH University of Krakow, al. A. Mickiewicza 30, 30-059 Krakow, Poland; 3 Department of Power Electronics and Energy Control Systems, Faculty of Electrical Engineering, Automatics, Computer Science and Biomedical Engineering, AGH University of Krakow, al. A. Mickiewicza 30, 30-059 Krakow, Poland; abien@agh.edu.pl

**Keywords:** electricity meter, measurement error, EMC, input circuits, current sensors, conducted disturbances

## Abstract

The influence of conducted electromagnetic disturbances on the energy measurement error of electricity meters remains insufficiently explored in electromagnetic compatibility (EMC) studies, particularly in the frequency range above the classical harmonic domain. The aim of this study was to investigate the susceptibility of electricity meters with different input configurations to conducted disturbances in the frequency range 1–150 kHz, including the 2–150 kHz band covered by IEC 61000-4-19. The novelty of this work lies in the comparative analysis of meters employing a shunt resistor, current transformer, Rogowski coil, Hall-effect sensor, and a digital system based on a Merging Unit and Sampled Values. The tests were performed as a preliminary screening stage of the study, using a test procedure based on IEC 61000-4-19, in which the energy measurement error was determined from the difference between the energy measured by the meter under test and that measured by a reference meter while disturbances were injected into the current circuit. The results showed that, for most of the tested electronic meters, the influence of the disturbances at the applied 1 A level was limited. For the tested Class B meters, the observed maximum error deviations remained below 1%, while the largest deviation under continuous-wave disturbance was observed for the Merging Unit + SV system. The highest immunity to amplitude-modulated disturbances was found for the shunt-based meter and the Rogowski coil-based meter. In none of the investigated cases were large error deviations on the order of several percent or several tens of percent observed. The obtained results indicate that, under the applied test conditions, conducted disturbances in the investigated frequency range did not cause significant deterioration of the metrological performance of most of the analyzed electronic meters. However, the results should be interpreted as a comparative assessment under modified IEC-based test conditions, rather than as a full compliance evaluation at the normative disturbance levels for directly connected meters.

## 1. Introduction

Electricity meters are a fundamental component of power system infrastructure, and the accuracy of their readings is of major importance both for commercial billing and for the assessment of grid operating conditions. Modern power networks are increasingly affected by power electronic devices, such as inverters, converters, switched-mode power supplies, and power-line communication systems [1,2]. These devices may generate conducted electromagnetic disturbances that can potentially affect the correct operation of metering systems.

Previous studies on the influence of disturbances on electricity meters have focused mainly on the effects of harmonics and interharmonics in the frequency range up to approximately 2–2.5 kHz [3,4,5,6,7,8]. The authors previously investigated several electricity meters with different input configurations in this frequency range and did not observe any exceedance of the permissible error limits under the application of disturbances within this band [3].

For this reason, further investigations were undertaken to assess the susceptibility of electricity meters to disturbances in the higher frequency range from 2 kHz to 150 kHz [1,2,4,5,6,7,8]. This frequency band remains relatively insufficiently explored, even though the corresponding test framework and the applicable meter-related requirements have been standardized in IEC and WELMEC documents [1,2,9,10,11,12,13,14,15,16]. In particular, IEC 61000-4-19 defines the general test method, the parameters of the applied disturbances, and the conditions for immunity testing against differential-mode currents occurring in this frequency range [10]. For electricity meters, the corresponding product standards and WELMEC guidance documents further specify the applicable test conditions [9,10,11,12,13,14,15,16].

The aim of this study is to investigate the effect of conducted electromagnetic disturbances in the frequency range 2–150 kHz on the energy measurement error of electricity meters with different input configurations. The work has a comparative nature and focuses on a preliminary screening assessment of meters with different current-channel designs under an IEC-based disturbance procedure. Particular attention was given to comparing the behavior of meters employing different current signal conditioning circuits.

The results showed that, for most of the analyzed meters, the influence of disturbances in this frequency range was limited and did not lead to substantial deterioration of metrological performance under the applied test conditions. A more pronounced susceptibility was observed only for the digital system based on a Merging Unit and energy measurement using Sampled Values.

## 2. Materials and Methods

### 2.1. Investigated Electricity Meters

Electricity meters with different input configurations in their current channels were selected for this study (Table 1). The analysis included directly connected Class B (1%) meters representing different input signal processing technologies, as well as a digital system composed of a Merging Unit and a meter using Sampled Values in accordance with IEC 61850-9-2 [17]. The selection of meters was intended to enable a comparison of susceptibility to conducted disturbances depending on the applied input circuit.

Additionally, an induction meter was tested, although this type of meter is formally outside the scope of IEC 61000-4-19; it was nevertheless included in the study in order to extend the comparative analysis.

The system composed of a Merging Unit and a meter using Sampled Values was available only during the continuous-wave disturbance tests. At the time the amplitude-modulated disturbance tests were carried out, this system was no longer available; therefore, it was not included in that part of the experiment.

### 2.2. Test Setup

A test setup was constructed in order to simulate electric power and to inject controlled current disturbances into the current circuit of the meter under test.

Electric power was simulated using a programmable power generator of the PGM-02/3 (IKSAiP, Wrocław, Poland) type, equipped with independent voltage and current channels. The generator allowed for independent adjustment of voltage, current, and the phase angle between them.

A reference meter, RD-22-231 (Radian Research, Inc., Lafayette, IN, USA), was included in the measurement system and served as the reference for determining the energy measurement error of the tested meter. The maximum relative error of the reference meter did not exceed 100 µWh/Wh.

The voltage circuits of the tested meter and the reference meter were connected in parallel and supplied with the same rated voltage. In the case of three-phase meters, the voltage was applied simultaneously to all phases. The current circuits of the meters were connected in series, ensuring that the same reference current flowed through both the reference meter and the meter under test.

A 2 Ω resistor was used to separate the reference current from the disturbance current. The reference current, the disturbance current, and their sum applied to the tested meter were monitored using a three-phase power quality analyzer, Yokogawa WT5000 (Yokogawa Test & Measurement Corporation, Hachioji, Tokyo, Japan). The entire setup was controlled using custom-developed LabView 2010 (National Instruments, Austin, TX, USA) software.

**Figure 1:**
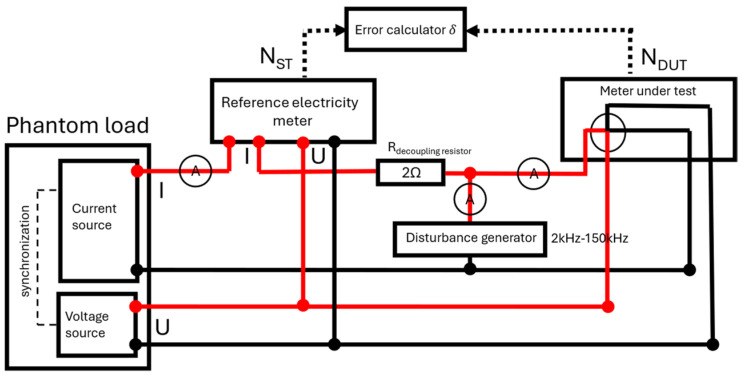
A schematic diagram of the test setup for determining electricity meter error characteristics under conducted disturbances (2–150 kHz). The red line indicates the phase conductor in the voltage and current circuits.; authors’ design.

A schematic diagram of the test setup is shown in Figure 1.

### 2.3. Disturbance Generation System

Disturbances were generated using an arbitrary waveform generator system built from two synchronized DDS (Direct Digital Synthesis) generators. The master generator was an Agilent 33250A (Agilent Technologies, Santa Clara, CA, USA), whereas the slave generator was a Hameg HM8131-2 (HAMEG Instruments GmbH, Mainhausen, Germany). The use of two generators made it possible to obtain amplitude-keyed waveforms while maintaining full time synchronization of the signals.

The disturbance signal was subsequently amplified using a PEA5C transconductance amplifier (Popek Elektronik, Zamość, Poland) transconductance amplifier, which enabled generation of the required disturbance current in the current circuit of the meter under test in accordance with the test conditions.

**Figure 2:**
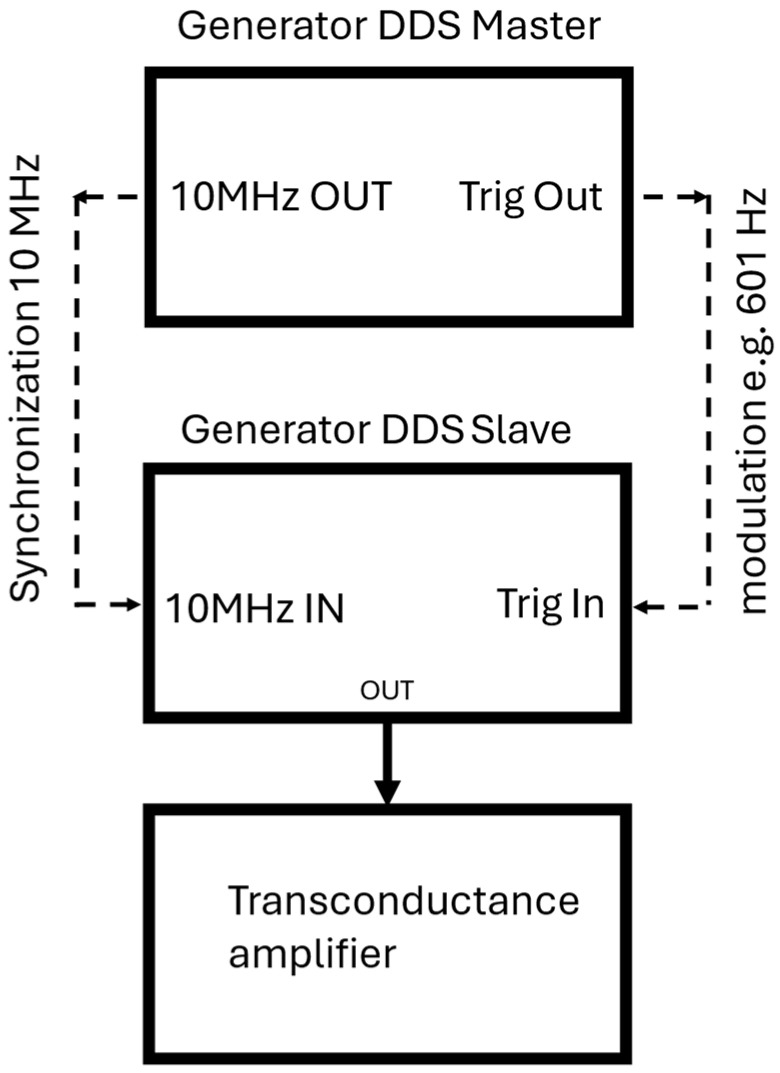
Arbitrary waveform generator based on two synchronized DDS generators used for generation of fully synchronized amplitude-keyed waveforms.

A schematic diagram of the disturbance generation system is shown in Figure 2.

Two types of disturbance waveforms were applied during the tests: a continuous wave (CW) in the frequency range from 2 kHz to 150 kHz, and a CW waveform amplitude-modulated by a rectangular signal [10]. The modulation frequencies were 3 Hz, 101 Hz, 301 Hz, and 601 Hz, respectively.

### 2.4. Disturbance Parameters and Test Conditions

The immunity of electricity meters to conducted differential-mode disturbances in the frequency range from 1 kHz to 150 kHz was investigated using a test procedure based on IEC 61000-4-19:2014 and the corresponding meter-related requirements [10,11,12,13,14,15]. The purpose of the test was to assess the effect of conducted electromagnetic disturbances, occurring in power networks mainly due to power electronic converters and power-line communication systems, on the energy measurement error of electricity meters [1,2,10]. During the tests, the disturbance was injected exclusively into the current circuit of the meter under test, whereas the voltage circuit remained free from disturbance [10].

The tests were performed under the following operating conditions:

- The voltage circuits were supplied with rated voltage;
- The current circuits carried the current specified for the relevant meter accuracy class;
- The power factor of the measured signal was set in accordance with the applicable accuracy-class requirements.

In the case of polyphase meters, the same voltage was applied to all three phases, whereas the current was applied only to the first current channel.

The differential disturbance current, $I_{diff}$, was injected into the current circuit of the meter under test. However, it should be emphasized that the present study did not constitute a full compliance test under all standardized conditions for directly connected electricity meters. Instead, it was designed as a preliminary screening stage of the study, intended to provide a comparative assessment of meters with different current channel configurations under identical excitation conditions.

For this reason, a disturbance current of 1 A was applied for both the continuous-wave and amplitude-modulated tests over the whole investigated frequency range. In the generic IEC 61000-4-19 framework, 1 A corresponds to Level 1 in the range 2–30 kHz, whereas the product-standard conditions for directly connected electricity meters specify higher levels, namely 3 A in the range 2–30 kHz and 1.5 A in the range 30–150 kHz. In addition, the investigated frequency range was extended from the standardized 2–150 kHz interval to 1–150 kHz, and the frequency sweep was performed with a step of 1% of the current frequency, providing a finer frequency resolution than that used in the standardized meter test conditions. Moreover, for the continuous-wave test, a CW waveform without pauses was applied. A comparison between the standardized test conditions for directly connected meters and the parameters applied in the present study is given in Table 2.

Two types of disturbance waveforms were used: a continuous wave (CW) and a rectangularly amplitude-modulated waveform. The modulation frequencies were 3 Hz, 101 Hz, 301 Hz, and 601 Hz, in accordance with the IEC-based rectangularly modulated pulse test procedure for a 50 Hz mains frequency [10].

The frequency sweep was carried out with a step equal to 1% of the current frequency. The first and the last measurements were performed without disturbance, at the fundamental frequency of 50 Hz. The first measurement served as the reference measurement, and the disturbance effect was subsequently evaluated with respect to this reference result. This approach reduced the influence of the setup components on the assessment of the disturbance effect itself on the meter under test.

The results should therefore be interpreted as a comparative assessment of disturbance effects under modified IEC-based test conditions, rather than as a full compliance evaluation against the normative product-standard levels for directly connected meters.

### 2.5. Recording of Waveforms in the Current Circuit

During the tests, the reference current, the disturbance current, and the resulting current applied to the meter under test were monitored simultaneously. The waveforms were recorded using a WT5000 power quality analyzer, which made it possible to verify the correct superposition of signals in the current circuit and to control the stability of the applied test conditions.

**Figure 3:**
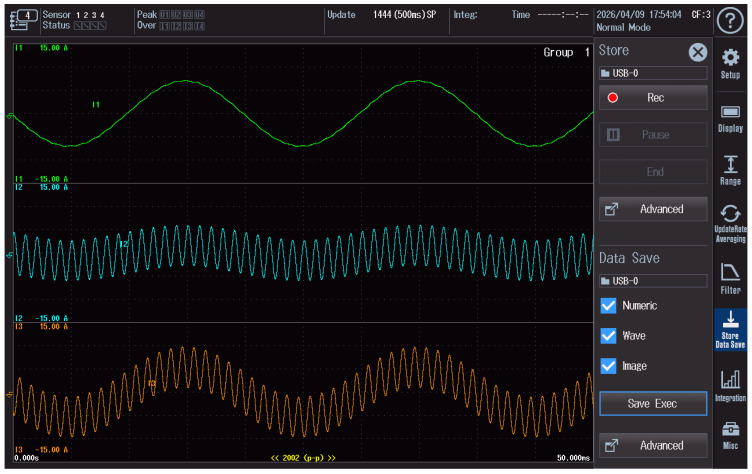
The waveforms of the reference current, disturbance current, and resulting current recorded during the continuous-wave (CW) test and exported directly from the WT5000 analyzer.

**Figure 4:**
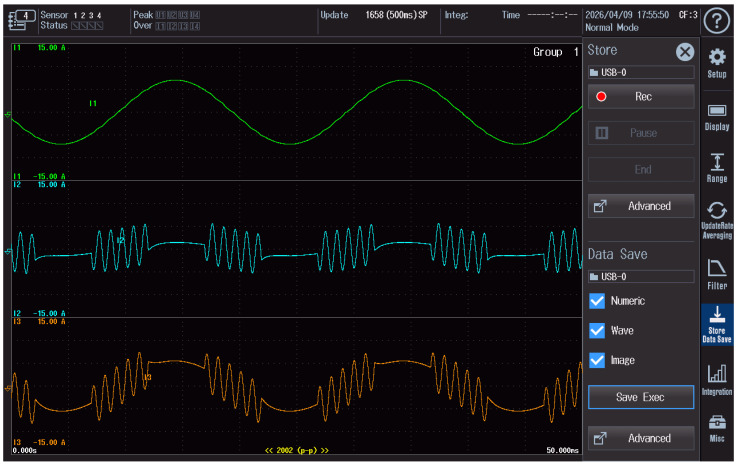
The waveforms of the reference current, disturbance current, and resulting current recorded during the rectangularly amplitude-modulated disturbance test and exported directly from the WT5000 analyzer.

Examples of waveforms recorded during the continuous-wave (CW) test are shown in Figure 3, whereas the waveform recorded during the rectangularly amplitude-modulated disturbance test is presented in Figure 4. In both figures, the green trace represents the reference current, the blue trace corresponds to the disturbance current, and the orange trace represents the resulting current supplied to the meter under test. The oscillograms were exported directly from the analyzer.

The values shown on the vertical axes, such as ±15 A, correspond to the measurement ranges of the analyzer channels rather than to the actual amplitudes of the measured currents. The recorded oscillograms are provided for illustrative purposes only, whereas the main quantitative results of the study are presented in Section 3.

### 2.6. Determination of Energy Measurement Error

The relative error of the meter under test was determined by comparing the energy measured by the tested meter with the energy measured by the reference meter. The relative error was calculated according to the following relationship:

$$\delta =100\%\frac{E_{DUT}-E_{REF}}{E_{REF}}$$

where:

- *δ*—relative energy measurement error [%];
- *E*_DUT_—energy measured by the meter under test;
- *E*_REF_—energy measured by the reference meter.

The reference energy was determined on the basis of the readings of the RD22 reference meter.

In order to quantify the effect of the disturbance itself, the change in error with respect to the reference measurement was also considered. For each test series, the first measurement was performed at 50 Hz without any disturbance and served as the reference measurement. The disturbance-induced change in error was then defined as follows:

$$\Delta \delta (f)=\delta_{dist}(f)-\delta_{ref}$$

where:

Δ*δ*(*f*)—change in the relative energy measurement error caused by the disturbance at frequency f;

*δ*_dist_(*f*)—relative error determined in the presence of a disturbance at frequency f;

*δ*_ref_—relative error determined under reference conditions without disturbance.

For comparative evaluation, the maximum absolute value of the disturbance-induced error change was used as a summary indicator:

$$\Delta \delta_{max}=max|\Delta \delta (f)|$$

This quantity was used to summarize the most unfavorable cases for the investigated meters and disturbance types in Table 3 and Table 4. The adopted approach made it possible to reduce the influence of the measurement setup itself on the interpretation of the disturbance effect and to compare the investigated meters on a common quantitative basis.

## 3. Results

### 3.1. Results for Continuous-Wave Disturbance

**Figure 5:**
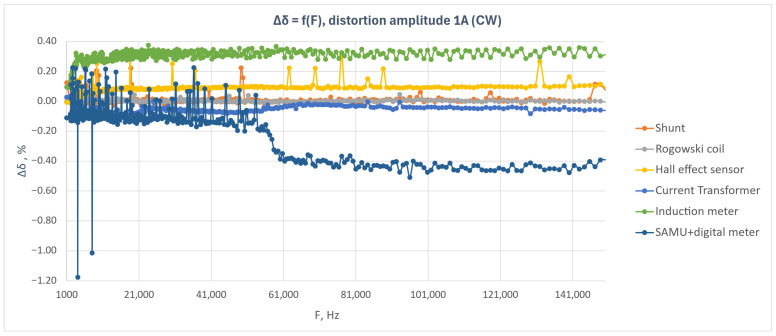
Relative energy measurement error as a function of disturbance frequency for the tested electricity meters during the continuous-wave test with a disturbance amplitude of 1 A.

Figure 5 shows the relative energy measurement error as a function of disturbance frequency for the six tested meters under a continuous-wave disturbance with an amplitude of 1 A.

The results presented in Figure 5 indicate that, for most of the tested meters, the influence of the continuous-wave disturbance in the frequency range from 2 kHz to 150 kHz was limited. The meters equipped with a shunt resistor, Rogowski coil, Hall-effect sensor, and current transformer exhibited relatively stable error characteristics throughout the analyzed frequency band. The variations in error were of small amplitude and, apart from isolated points, did not indicate any significant deterioration in the metrological performance of these devices.

The induction meter exhibited a positive error that remained at a similar level over the entire investigated frequency range. The observed characteristic suggests that, in this case, the disturbance did not cause any significant change in the frequency response of the meter, and the observed error level can be regarded as an approximately constant offset.

The highest sensitivity to the disturbance was observed for the system composed of a SAMU module and a meter using Sampled Values. In the lower part of the frequency range, a larger scatter of results was visible, whereas above approximately 55–60 kHz a distinct change in the error level occurred and persisted with further increase in frequency. This result indicates a greater susceptibility of the digital metering chain to conducted disturbances in the upper part of the analyzed band.

Local error peaks are also visible in the plot for selected meters, particularly in the lower frequency range. During the tests, it was found that this effect could be reduced by increasing the number of pulses recorded by the reference meter and by performing a larger number of repeated measurements for a given frequency. However, this approach leads to a significant increase in the total duration of the experiment.

The obtained results indicate that, for five out of the six analyzed systems, the continuous-wave disturbance with an amplitude of 1 A did not cause any significant increase in the energy measurement error. The largest deviations were observed for the SAMU + SV meter system, particularly in the frequency range above approximately 50 kHz.

For amplitude-modulated disturbances, five meters were analyzed: a shunt-based meter, a current-transformer-based meter, a Rogowski coil-based meter, a Hall sensor-based meter, and an induction meter. The Merging Unit + Sampled Values system was not included in this part of the study because it was no longer available at the time the tests were performed.

To facilitate comparison among the investigated meters, the maximum observed absolute error deviations for the continuous-wave disturbance test are summarized in Table 3.

As shown in Table 3, the largest deviation under continuous-wave disturbance was observed for the MU + SV system, whereas the remaining investigated electronic meters exhibited clearly lower maximum deviations. For the conventional meters, the observed maximum deviations remained within the limits corresponding to their declared 1% accuracy class.

### 3.2. Results for the Shunt-Based Meter Under Amplitude-Modulated Disturbances

**Figure 6:**
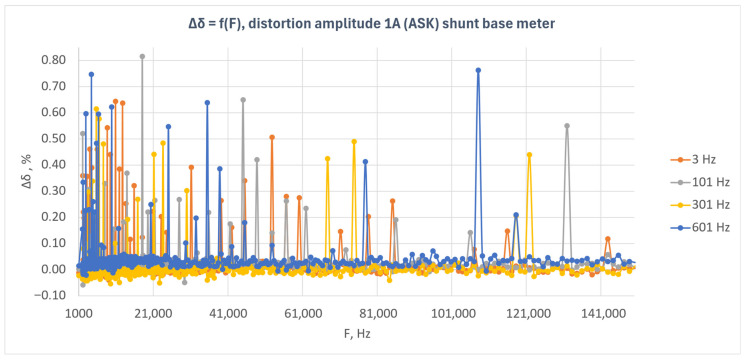
Relative energy measurement error as a function of disturbance frequency for the shunt-based meter under amplitude-modulated disturbances (ASK) with a disturbance amplitude of 1 A.

Figure 6 presents the relative energy measurement error as a function of disturbance frequency for the shunt-based meter under amplitude-modulated disturbances (ASK). The results are shown for four modulation frequencies: 3 Hz, 101 Hz, 301 Hz, and 601 Hz.

The results presented in Figure 6 indicate that the shunt-based meter exhibited high immunity to amplitude-modulated disturbances over the entire investigated frequency range. For most measurement points, the error values remained close to zero, and the observed deviations were small.

For all analyzed modulation frequencies, however, isolated local error peaks are visible, particularly in the lower part of the investigated frequency band. The largest number of such deviations occurred for modulation frequencies of 3 Hz and 101 Hz, whereas for 301 Hz and 601 Hz the characteristics were more compact, despite the presence of isolated outlying points also in the higher frequency ranges.

The observed local maxima do not form continuous regions of increased error but rather have a point-like nature. This indicates that they are not associated with a permanent loss of the meter’s metrological immunity, but rather with temporary instability of the measurement result under specific frequency conditions. During the tests, it was found that this effect could be reduced by increasing the number of pulses recorded by the reference meter and by performing a larger number of repetitions for a given frequency. However, this leads to a significant increase in the total duration of the experiment.

The analysis of the results indicates that, in the case of the shunt-based meter, amplitude-modulated disturbances with an amplitude of 1 A did not cause any systematic increase in the energy measurement error as a function of frequency. The meter response was stable, and the observed deviations were mainly local and incidental.

### 3.3. Results for the Current Transformer-Based Meter Under Amplitude-Modulated Disturbances

**Figure 7:**
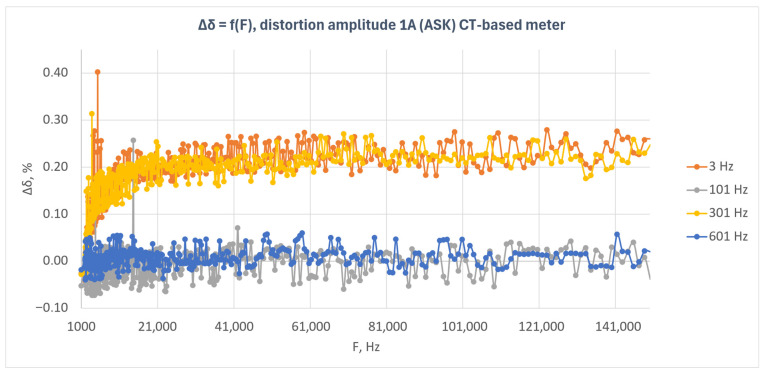
Relative energy measurement error as a function of disturbance frequency for the current-transformer-based meter under amplitude-modulated disturbances (ASK) with a disturbance amplitude of 1 A.

Figure 7 presents the relative energy measurement error as a function of disturbance frequency for the current transformer-based meter under amplitude-modulated disturbances (ASK). The characteristics obtained for four modulation frequencies—3 Hz, 101 Hz, 301 Hz, and 601 Hz—are shown.

The results presented in Figure 7 indicate that the response of the current-transformer-based meter depended on the modulation frequency of the disturbance signal. The highest error values were observed for modulation frequencies of 3 Hz and 301 Hz. In both cases, the error assumed positive values and, after an initial increase, stabilized at a level of approximately 0.20–0.25% over a wide range of disturbance frequencies.

For modulation frequencies of 101 Hz and 601 Hz, the characteristics exhibited a substantially lower level. The error values remained close to zero and showed only small fluctuations throughout the analyzed frequency band. This indicates that, for these modulation frequencies, the disturbance did not cause any significant shift in the metrological characteristic of the tested meter.

Compared with the shunt-based meter, the response of this system exhibits a more ordered pattern. Instead of isolated, randomly distributed peaks, a clear separation of the characteristics corresponding to the individual modulation frequencies can be observed. This suggests that, in the current transformer-based meter, the effect of amplitude-modulated disturbances depends not only on the carrier frequency but also on the modulation frequency.

At the beginning of the investigated frequency range, particularly near the lower band limit, a larger scatter of results and isolated outlying points can be observed. At higher frequencies, the characteristics become more stable. This may indicate that the greatest instability of the system response occurs in the lower part of the analyzed frequency range.

The obtained results indicate that the current transformer-based meter maintained immunity to amplitude-modulated disturbances; however, the error level was clearly dependent on the modulation frequency. The strongest influence of the disturbance was observed for modulation frequencies of 3 Hz and 301 Hz, whereas for 101 Hz and 601 Hz the effect remained limited.

### 3.4. Results for the Rogowski Coil-Based Meter Under Amplitude-Modulated Disturbances

**Figure 8:**
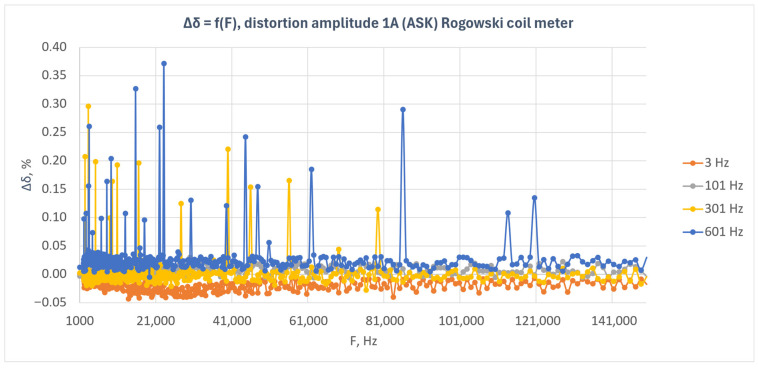
Relative energy measurement error as a function of disturbance frequency for the Rogowski coil-based meter under amplitude-modulated disturbances (ASK) with a disturbance amplitude of 1 A.

Figure 8 presents the relative energy measurement error as a function of disturbance frequency for the Rogowski coil-based meter under amplitude-modulated disturbances (ASK). The results obtained for four modulation frequencies—3 Hz, 101 Hz, 301 Hz, and 601 Hz—are shown.

The results presented in Figure 8 indicate that the Rogowski coil-based meter exhibited low sensitivity to amplitude-modulated disturbances over the entire investigated frequency range. For most measurement points, the error values remained close to zero, and the observed deviations were small and did not show any clear trend with increasing disturbance frequency.

The smallest deviations were observed for the 101 Hz modulation frequency, for which the characteristic remained practically centered around zero. For the 3 Hz modulation, the characteristic showed a slightly negative offset that remained at a similar level over the entire frequency band. In the case of 301 Hz and 601 Hz modulation, isolated positive error peaks were observed, with both the number and amplitude of these peaks being greater for 601 Hz.

The observed local maxima were point-like in nature and did not form continuous regions of increased error. This indicates that the response of the Rogowski coil-based meter was generally stable and that the observed deviations were incidental. In particular, for the 601 Hz modulation frequency, isolated points with larger error values were visible in different parts of the investigated frequency band; however, they did not lead to a permanent shift of the entire characteristic.

In contrast to the current transformer-based meter, no clear separation of the characteristics corresponding to the individual modulation frequencies can be observed in this case. The response of the Rogowski coil-based meter is more compact, and the effect of the modulation frequency is manifested mainly through differences in the number and amplitude of isolated error peaks.

The obtained results indicate that the Rogowski coil-based meter maintained good immunity to amplitude-modulated disturbances with an amplitude of 1 A. The influence of the disturbance on the energy measurement error was limited, and the observed deviations were mainly local in nature.

### 3.5. Results for the Hall Sensor-Based Meter Under Amplitude-Modulated Disturbances

**Figure 9:**
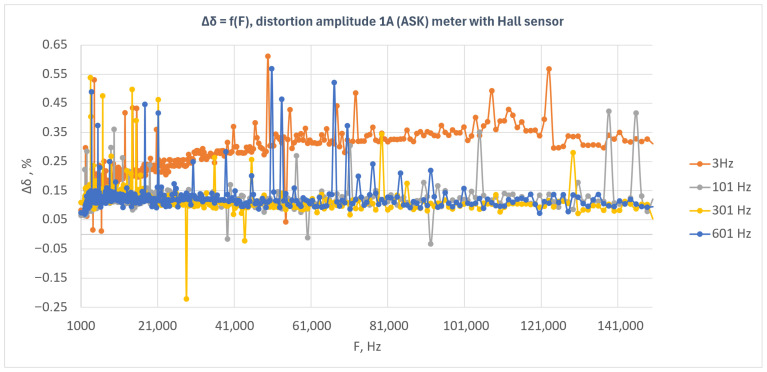
Relative energy measurement error as a function of disturbance frequency for the Hall sensor-based meter under amplitude-modulated disturbances (ASK) with a disturbance amplitude of 1 A.

Figure 9 presents the relative energy measurement error as a function of disturbance frequency for the Hall sensor-based meter under amplitude-modulated disturbances (ASK). The results obtained for four modulation frequencies—3 Hz, 101 Hz, 301 Hz, and 601 Hz—are shown.

The results presented in Figure 9 indicate that the Hall sensor-based meter exhibited a positive error over the entire investigated frequency range, with the error level depending on the modulation frequency of the disturbance signal. The highest error values were observed for the 3 Hz modulation frequency, for which the characteristic showed a clear increasing trend as a function of disturbance frequency and then stabilized at approximately 0.30–0.35%. This indicates that, for this modulation frequency, the meter was the most susceptible to the applied disturbance.

For modulation frequencies of 101 Hz, 301 Hz, and 601 Hz, the error level was clearly lower and generally remained close to 0.10–0.15%. The characteristics corresponding to these frequencies were similar to one another, although isolated local deviations, both positive and negative, were observed. The most compact characteristic was obtained for 601 Hz, whereas for 101 Hz and 301 Hz sporadic outlying points of larger amplitude were present.

In contrast to the Rogowski coil-based meter and the shunt-based meter, a clear separation of the characteristic corresponding to the 3 Hz modulation frequency from the remaining curves can be observed in this case. This indicates that, for the Hall sensor-based meter, the modulation frequency had a significant effect on the energy measurement error, with the highest susceptibility occurring for the lowest analyzed modulation frequency.

The plot also shows isolated local error peaks occurring in different parts of the investigated band. However, these peaks do not form continuous regions of instability and are rather point-like in character. This phenomenon may be associated with the limited number of pulses recorded during a single measurement and with the variability in system response at specific frequency points.

The obtained results indicate that the Hall sensor-based meter maintained immunity to amplitude-modulated disturbances with an amplitude of 1 A; however, the system response was clearly dependent on the modulation frequency. The greatest influence of the disturbance was observed for the 3 Hz modulation, whereas for the remaining modulation frequencies the error level remained lower and more stable.

### 3.6. Results for the Induction Meter Under Amplitude-Modulated Disturbances

It should be noted that the induction meter is outside the scope of the test specified in IEC 61000-4-19. Nevertheless, it was additionally examined in order to compare its response to disturbances with that of electronic meters.

**Figure 10:**
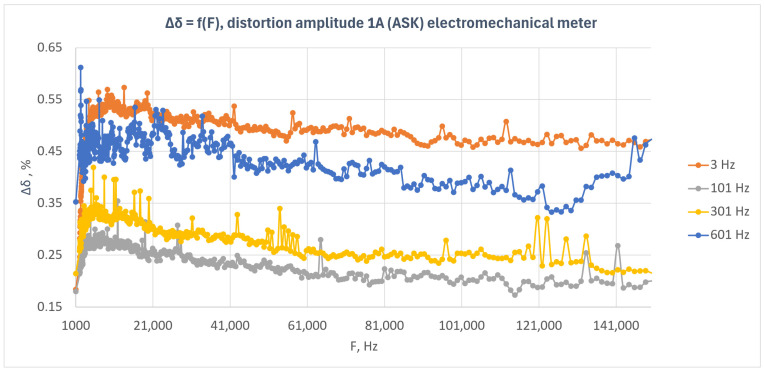
Relative energy measurement error as a function of disturbance frequency for the induction meter under amplitude-modulated disturbances (ASK) with a disturbance amplitude of 1 A.

Figure 10 presents the relative energy measurement error as a function of disturbance frequency for the induction meter under amplitude-modulated disturbances (ASK). The results obtained for four modulation frequencies—3 Hz, 101 Hz, 301 Hz, and 601 Hz—are shown.

The results presented in Figure 10 indicate that the induction meter exhibited a positive error over the entire investigated frequency range, with the level of this error being clearly dependent on the modulation frequency of the disturbance signal. In contrast to the previously analyzed electronic meters, all characteristics in this case remained clearly above zero and formed distinct error levels.

The highest error values were observed for the 3 Hz modulation frequency, for which the error generally remained at approximately 0.46–0.52% over a wide range of disturbance frequencies. Slightly lower values were obtained for the 601 Hz modulation, where the characteristic remained mainly within the range of approximately 0.35–0.47%. For the 301 Hz modulation, the error level was lower and was approximately 0.22–0.32%, whereas the smallest values were obtained for the 101 Hz modulation, for which the error generally remained within the range of approximately 0.18–0.27%.

The characteristics for all modulation frequencies had a relatively ordered course and showed only small local fluctuations. In many cases, a slight decreasing trend in the error value with increasing disturbance frequency can be observed, particularly for the 101 Hz, 301 Hz, and 601 Hz modulations. At the same time, the individual characteristics remain clearly separated from one another, indicating a strong dependence of the meter response on the modulation frequency.

Although isolated local peaks are present in the plot, they do not dominate the overall character of the response. In contrast to some of the electronic meters, for which mainly point-like deviations around values close to zero were observed, the induction meter exhibits a persistent shift in the error level that depends on the modulation frequency of the disturbance.

The obtained results indicate that the induction meter was more susceptible to amplitude-modulated disturbances than the tested electronic meters. The influence of the disturbance was systematic in nature, and the error level remained significantly dependent on the modulation frequency over the entire investigated frequency range.

### 3.7. Comparison of Results for Amplitude-Modulated Disturbances

A comparison of the results obtained for amplitude-modulated disturbances indicates that the response of the tested meters varied both in terms of error level and in terms of dependence on modulation frequency. The highest immunity was exhibited by the shunt-based meter and the Rogowski coil-based meter. In both cases, the error values generally remained close to zero, and the observed deviations were mainly in the form of local peaks.

The current transformer-based meter showed a more ordered dependence on modulation frequency. For modulation frequencies of 3 Hz and 301 Hz, a positive error level was observed over a wide frequency range, whereas for 101 Hz and 601 Hz the error values remained close to zero. This indicates that, in this case, the influence of the disturbance was clearly dependent on the modulation frequency.

The Hall sensor-based meter also exhibited a dependence on modulation frequency, with the greatest influence of the disturbance observed for the 3 Hz modulation. For the remaining modulation frequencies, the error level was lower and more stable.

The additionally tested induction meter exhibited the highest and most systematic error level among all analyzed systems. In this case, the influence of the disturbance had a clearly ordered character, and the error level remained strongly dependent on the modulation frequency.

Based on the obtained results, it can be concluded that, among the electronic meters, the highest immunity to amplitude-modulated disturbances was exhibited by the shunt-based and Rogowski coil-based configurations, whereas a stronger dependence on modulation frequency was observed for the current transformer-based meter and the Hall sensor-based meter. For rectangular-modulated disturbances, the maximum observed absolute error deviations for the individual meters and modulation frequencies are summarized in Table 4.

Table 4 confirms that the shunt-based and Rogowski coil-based meters exhibited the lowest overall susceptibility, whereas larger maximum deviations were observed for the Hall sensor-based meter and the induction meter. However, for all conventional meters, the observed maximum deviations still remained within the limits corresponding to their declared 1% accuracy class.

## 4. Discussion

The obtained results indicate that the influence of conducted electromagnetic disturbances on the energy measurement error depends on the current channel configuration of the investigated meter; however, under the applied test conditions, this influence was generally limited. For most of the tested electronic meters, the observed error variations remained small and did not indicate substantial deterioration of metrological performance. The most stable response to amplitude-modulated disturbances was observed for the shunt-based meter and the Rogowski coil-based meter, whereas the current transformer-based meter and the Hall sensor-based meter exhibited a more pronounced dependence of error on modulation frequency. The greatest susceptibility to continuous-wave disturbance was observed for the Merging Unit + SV system.

The results should be interpreted in the context of the applied methodology and the adopted test scope. The study was designed as a preliminary screening stage intended to compare meters with different current-channel configurations under identical excitation conditions. A disturbance current of 1 A was applied over the whole investigated frequency range, which was lower than the product-standard levels specified for directly connected meters in the relevant meter standards. In addition, the test conditions differed from the standardized meter-compliance procedure with respect to the applied voltage level, the frequency step, the investigated frequency range, and the continuous-wave profile. Therefore, the obtained results should be regarded as a comparative assessment under modified IEC-based test conditions rather than as a full compliance evaluation against the normative disturbance levels.

It should also be emphasized that the purpose of the present study was not to diagnose the internal mechanisms of the individual input circuits, but rather to compare the metrological behavior of meters with different current channel designs under controlled disturbance conditions. Since the observed deviations were generally small, attributing them unambiguously to specific physical mechanisms associated with the individual sensor principles would be overly speculative. At least part of the local deviations may be related to measurement scatter and methodological limitations rather than to clear structural differences between the sensing elements themselves. In this sense, the main outcome of the study is that, under the applied disturbance conditions, no large metering errors of the type reported in some earlier research were observed. Further work should include a dedicated mechanistic analysis of disturbance effects with respect to the operating principles of the individual input circuits, in particular the role of magnetic components, active electronic front ends, analog and digital filtering, and signal processing stages in digital metering chains.

A further limitation of the study is that the MU + SV system was available only during the continuous-wave tests. As a result, its susceptibility to amplitude-modulated disturbances could not be assessed, which limits the completeness of the comparison among all investigated systems. Consequently, the conclusions regarding the digital system apply only to the continuous-wave disturbance case.

## 5. Conclusions

This study investigated the influence of conducted electromagnetic disturbances in the frequency range 1–150 kHz on the energy measurement error of electricity meters with different input configurations. The performed tests showed that, for most of the analyzed electronic meters, the influence of the disturbances was limited under the applied test conditions. The highest immunity to amplitude-modulated disturbances was observed for the shunt-based meter and the Rogowski coil-based meter, whereas the greatest susceptibility to continuous-wave disturbance was found for the Merging Unit + SV system. In none of the investigated cases were large error deviations on the order of several percent or several tens of percent observed.

The obtained results indicate that, under the applied disturbance conditions, conducted disturbances in the investigated frequency range did not cause significant deterioration in the metrological performance of most of the analyzed electronic meters. However, the results should be interpreted as a comparative assessment under modified IEC-based test conditions rather than as a full compliance evaluation at the normative disturbance levels for directly connected meters.

It should also be noted that the Merging Unit + SV system was not available during the amplitude-modulated tests; therefore, the conclusions regarding this system apply only to the continuous-wave tests. Further studies should focus primarily on digital metering systems and their behavior under amplitude-modulated disturbances. A dedicated mechanistic analysis of disturbance propagation in different meter input circuits, including shunt-, transformer-, Rogowski coil-, Hall sensor-, and digital-based measurement chains, should also be considered an important direction for future work.

## Figures and Tables

**Table 1:** Electricity meters investigated in study and their input current sensing methods.

Meter Type	Current Sensing Method	Accuracy Class	Nominal Current, A	Measurement Principle
Static meter	Shunt resistor	Class B (1%)	5	Direct measurement
Static meter	Current transformer (CT)	Class B (1%)	5	Magnetic transformer
Static meter	Rogowski coil	Class B (1%)	5	Air-core current sensor
Static meter	Hall effect sensor	Class B (1%)	5	Magnetic field sensing
Induction meter	Electromechanical system	Class B (1%)	5	Ferraris principle
Digital meter + SAMU	Sampled Values (IEC 61850-9-2)	Class 0.2	5	Digital measurement chain

**Table 2:** Comparison of the standardized test conditions for directly connected electricity meters and the parameters applied in this study.

Parameter	Standardized Value for Directly Connected Meters	Applied in This Study	Comment
Frequency range	2–150 kHz	1–150 kHz	Extended frequency range Finer frequency resolution; value adopted from IEC 62052-11:2020 [11]
Frequency step	2%	1%	
Disturbance type	Differential current	Differential current	No deviation
Current level, 2–30 kHz	3 A	1 A	Equal to IEC 61000-4-19 Level 1; lower than the product-standard level [10]
Current level, 30–150 kHz	1.5 A	1 A	Higher than IEC 61000-4-19 Level 1; lower than the product-standard level [10]
Disturbance signal profiles	CW with pauses; rectangularly modulated pulses	CW without pauses; rectangularly modulated pulses	Modified test condition used for a preliminary screening stage
Modulation frequencies	3 Hz, 101 Hz, 301 Hz, 601 Hz	3 Hz, 101 Hz, 301 Hz, 601 Hz	No deviation
Disturbance current tolerance	±5%	±5%	No deviation
Voltage conditions	Minimum specified voltage	Rated voltage	Modified test condition used for a preliminary screening stage

**Table 3:** Maximum observed error deviations for continuous-wave disturbances.

Meter/System	Input Configuration	Disturbance Type	Maximum Absolute Error Deviation Δ*δ*_max_, %	Frequency of Maximum Deviation, Hz
Shunt-based meter	Shunt resistor	CW, 1 A	0.224	49,262
CT-based meter	Current transformer	CW, 1 A	0.156	13,246
Rogowski coil-based meter	Rogowski coil	CW, 1 A	0.122	2299
Hall sensor-based meter	Hall sensor	CW, 1 A	0.335	18,579
Induction meter	Electromechanical	CW, 1 A	0.375	23,590
MU + SV system	Digital metering chain	CW, 1 A	1.176	4054

**Table 4:** Maximum observed error deviations for rectangular-modulated disturbances.

Meter/System	Input Configuration	Modulation Frequency, Hz	Maximum Absolute error Deviation Δ*δ*_max_, %	Frequency of Maximum Deviation, Hz
Shunt-based meter	Shunt resistor	3	0.644	10,856
Shunt-based meter	Shunt resistor	101	0.816	18,032
Shunt-based meter	Shunt resistor	301	0.763	108,119
Shunt-based meter	Shunt resistor	601	0.615	5686
CT-based meter	Current transformer	3	0.402	5303
CT-based meter	Current transformer	101	0.257	14,632
CT-based meter	Current transformer	301	0.314	3781
CT-based meter	Current transformer	601	0.060	58,925
Rogowski coil-based meter	Rogowski coil	3	0.043	13,784
Rogowski coil-based meter	Rogowski coil	101	0.030	29,953
Rogowski coil-based meter	Rogowski coil	301	0.296	3257
Rogowski coil-based meter	Rogowski coil	601	0.372	23,125
Hall sensor-based meter	Hall sensor	3	0.612	49,755
Hall sensor-based meter	Hall sensor	101	0.423	138,655
Hall sensor-based meter	Hall sensor	301	0.538	3492
Hall sensor-based meter	Hall sensor	601	0.569	50,755
Induction meter	Electromechanical	3	0.573	13,512
Induction meter	Electromechanical	101	0.354	11,873
Induction meter	Electromechanical	301	0.419	5574
Induction meter	Electromechanical	601	0.612	2254

## Data Availability

The data presented in this study are available from the corresponding author upon reasonable request.

## Abbreviations

The following abbreviations are used in this manuscript:

ASK	Amplitude Shift Keying
CT	Current Transformer
CW	Continuous Wave
DDS	Direct Digital Synthesis
DUT	Device Under Test
EMC	Electromagnetic Compatibility
IEC	International Electrotechnical Commission
MU	Merging Unit
SAMU	Stand-Alone Merging Unit
SV	Sampled Value

## References

[1] Mariscotti A., Mingotti A. (2024). The Effects of Supraharmonic Distortion in MV and LV AC Grids. Sensors.

[2] Wasowski M., Sikorski T., Wisniewski G., Kostyla P., Szymanda J., Habrych M., Gornicki L., Sokol J., Jurczyk M. (2021). The Impact of Supply Voltage Waveform Distortion on Non-Intentional Emission in the Frequency Range 2–150 kHz: An Experimental Study with Power-Line Communication and Selected End-User Equipment. Energies.

[3] Sadkowski G., Bień A. (2024). Analiza wpływu składowych harmonicznych na pomiar energii licznikiem elektronicznym—Studium przypadku. Prz. Elektrotech..

[4] Leferink F.B.J., Keyer C.H.A., Melentjev A. (2017). Static Energy Meter Errors Caused by Conducted Electromagnetic Interference. IEEE Electromagn. Compat. Mag..

[5] ten Have B., Hartman T., Moonen N., Leferink F. (2019). Misreadings of Static Energy Meters due to Conducted EMI Caused by Fast Changing Current. 2019 Joint International Symposium on Electromagnetic Compatibility, Sapporo and Asia-Pacific International Symposium on Electromagnetic Compatibility (EMC Sapporo/APEMC).

[6] Hartman T.H.F., ten Have B., Dijkstra J., Grootjans R., Moonen N., Leferink F. (2022). Susceptibility of Static Energy Meters due to Amplifier Clipping Caused by a Rogowski Coil. IEEE Trans. Electromagn. Compat..

[7] Kosobudzki G., Szafrańska M. (2024). Badania odporności liczników energii elektrycznej na zaburzenia elektromagnetyczne. Prz. Elektrotech..

[8] Szafrańska M., Kosobudzki G., Jóskiewicz Z. (2025). Immunity Tests of Static Electricity Meters to Additional Electromagnetic Disturbances. Prz. Elektrotech..

[9] WELMEC (2020). WELMEC Guide 11.1: Measuring Instruments Directive 2014/32/EU—Issue 8: General and Administrative Aspects of the Voluntary System of Modular Evaluation of Measuring Instruments.

[10] (2014). Electromagnetic Compatibility (EMC)—Part 4-19: Testing and Measurement Techniques—Test for Immunity to Conducted, Differential Mode Disturbances and Signalling in the Frequency Range 2 kHz to 150 kHz at A.C. Power Ports.

[11] (2020). Electricity Metering Equipment—General Requirements, Tests and Test Conditions—Part 11: Metering Equipment.

[12] (2020). Electricity Metering Equipment—Particular Requirements—Part 21: Static Meters for AC Active Energy (Classes 0.5, 1 and 2).

[13] (2020). Electricity Metering Equipment—Particular Requirements—Part 22: Static Meters for AC Active Energy (Classes 0.1S, 0.2S and 0.5S).

[14] (2020). Electricity Metering Equipment—Particular Requirements—Part 23: Static Meters for AC Reactive Energy (Classes 2 and 3).

[15] (2020). Electricity Metering Equipment—Particular Requirements—Part 24: Static Meters for AC Reactive Energy at Fundamental Frequency (Classes 0.5S, 1S, 1, 2 and 3).

[16] WELMEC (2025). WELMEC Guide 11.7: Modular Evaluation of Active Electrical Energy Meters.

[17] (2020). Communication Networks and Systems for Power Utility Automation—Part 9-2: Specific Communication Service Mapping (SCSM)—Sampled Values over ISO/IEC 8802-3.

